# Hospitals, Their Institution, Management, Etc.

**Published:** 1880-03

**Authors:** 


### EDITORIAL DEPARTMENT.

"If thou hast truth to utter, speak;
And leave the rest to God."

Hospitals, Their Institution, Management, etc.—The touch of Nature that makes all mankind akin, has doubtless exerted its influence commensurate with the existence of the higher types of the genus homo; and when it receives a responsive echo in a generous breast, results in deeds almost divine in their attributes and offices for good. In no other way, perhaps, have its benign and beautiful characteristics been so advantageously and strikingly displayed in deeds of benevolence and mercy as in the care and attention to the indigent sick and infirm of the races of men.

To retrospect, and recount the "labor of love," and the eleemosynary bestowals of the present century even, would occupy many folios, and we cannot, therefore, expect to do more than merely direct attention to a few prominent points in connection with the general subject of hospitals at this time. We hope that in doing this, however, to cast a few crumbs upon the waters of public attention that may be usefully gathered in the early future.

The location of a hospital for the treatment of disease is a matter of great importance. The site to be chosen should combine many necessary requisites: among them elevation, so as to insure proper drainage; isolation, to secure, as far as possible, freedom from noise and confusion; and ventilation, in order that the apartments may be kept in the best sanitary condition possible of attainment. The wards should be roomy and high, and supplied with proper facilities for freshening and purifying the air in them in winter as well as in the summer; and pure, fresh water should be had in abundance for drinking, culinary, washing and cleansing purposes. In a word, the location should be salubrious and healthy, with the requisites named, and all the adjuvants appertaining to them that modern science has been able to bestow. To these, of course, a fund should be supplied, sufficient to meet the wants and requirements of such an institution; and then, to complete the whole arrangement of a public hospital, a sufficient staff of well-trained and humane nurses, and a corps of intelligent, thoroughly educated and Christian physicians and surgeons should be added. These latter are demanded by the civilization of the age and the Christian country in which we live. The tendency of some who have been thrown to the surface by adventitious or fortuitous circumstances, is to ignore, or, at least, attempt to treat religious teaching and practice lightly and triflingly in such institutions, when clothed with the little authority that accident may have given them. This should not be permitted to take deeper root, for if there is one time more than another when poor, suffering humanity derives greater comfort than at any other from the example and teaching of the lowly Nazarene, it is when it is prostrated on a couch of disease and suffering in a charity hospital. The sufferer's condition places him entirely at the mercy of his physicians and nurses in such cases. How necessary, then, that these should practice and teach, as occasion might require, the great truths of the atonement and a judgment to come! Otherwise, the grand old name of Hotel Dieu has no significance at all. It is our purpose to revert to this matter again, and again, should it become necessary to awaken public attention to the state of affairs in places requiring it to be done. Let the following story of a case that has been told to us more than once suffice for the present to direct attention to the important matters alluded to above, viz.: A "professor" who was, and probably is still, the head medical attendant of a hospital founded by state aid, who seems not to have measured up to the standard we have named, diagnosed the case of a poor woman in its wards to be disease of the ovary, and invited quite a number of physicians and medical students to witness his maiden operation for ovariotomy. On the day and hour designated these assembled at the hospital, the latter anxious to profit by the grand achievement that had been promised them in gynecological surgery; and all things being arranged, the poor woman was placed in position, and incisions were made by the "professor," when, alas for the hopes of the operator, and, we might add, for his reputation also, as a diagnostician, a gush of serous fluid told the visitors in unmistakable language that ascites, and not ovarian disease, was the trouble with the patient. One of the visitors, who is an anatomist, it is said, quietly dissuaded the "professor" from further mutilation of his victim; and in a few hours she passed to that bourne from whence no patients return to trouble unhappy doctors. This is the story as it has been told to us.

